# Sudden Onset of Unexplained Leg Pain Following Open Reduction and Internal Fixation of a Displaced Tibial Tuberosity Fracture: A Case Report of Deep Venous Thrombosis in an Adolescent Athlete

**DOI:** 10.7759/cureus.76019

**Published:** 2024-12-19

**Authors:** Mustafa Fakih, Saleh Alfaisali, Reggie C Hamdy

**Affiliations:** 1 College of Medicine, McGill University, Montreal, CAN; 2 Pediatric Orthopedic Surgery, Shriners Hospitals for Children, Montreal, CAN; 3 Orthopedic Surgery, McGill University Health Centre, Montreal, CAN

**Keywords:** adolescent athlete, deep venous thrombosis (dvt), orthopaedic surgery, pediatric anticoagulation, pediatric orthopedic surgery, pediatric thromboprophylaxis, postoperative complications, tibial tuberosity fracture, venous doppler ultrasound, venous thromboembolism (vte)

## Abstract

This case report presents a 16-year-old basketball player, who developed deep venous thrombosis (DVT) following surgical intervention for a displaced tibial tuberosity fracture and forearm fractures. Despite few identifiable thrombotic risk factors, the patient’s postoperative course was complicated by unexplained leg pain, fever, and ultimately confirmed DVT. Prompt management with therapeutic anticoagulation and multidisciplinary care led to favourable outcomes. High-risk scores and diagnostic challenges underscore the complexity of anticipating a DVT in paediatric orthopaedic patients. This case highlights the importance of clinical vigilance in recognizing and managing DVT in young athletes undergoing orthopaedic surgery. Further research is needed to refine risk stratification and treatment guidelines for paediatric patients to mitigate the potential morbidity of postoperative DVT.

## Introduction

Development of a deep venous thrombosis (DVT) post-operation is a well-recognized, highly morbid adverse outcome, especially in older patients undergoing major surgeries. DVTs following pediatric surgeries on the other hand are more rare. Pediatric surgical patients found only 0.10% experienced a postoperative venous thromboembolism (VTE) event [1]. VTE/DVT risk factors in pediatric surgical patients are many, including but not limited to immobilization, surgery, ICU stay, oral contraceptive use, infection, and age; however, most are non-specific, making it difficult to anticipate an event before it occurs [1-3].

Pediatric orthopedic surgery patients are seen to have an increased risk of DVT compared to other hospitalized pediatric patients, especially those between the ages of 15 and 17, with one study showing a prevalence ratio of 2.2 for orthopedic patients [4]. Pediatric orthopedic trauma surgery, in particular, is associated with an even higher rate of VTE; a recent meta-analysis presented an incidence of 29 out of 10,000, compared to 14 for elective orthopedics surgeries [5].

Adolescents are at a crucial developmental stage marked by rapid growth, impacting their skeletal biomechanics [6]. This poses unique challenges for adolescent athletes, as they must navigate growth alongside the intense physical demands of their sports, particularly in contact sports, significantly elevating injury risks, and possibly needing surgical intervention [7-9].

There is currently no anticoagulant drug approved for use in children, with little research focusing on this topic and most data being extrapolated from adult studies. There are published safety data for the use of unfractionated heparin, low-molecular-weight heparin, fondaparinux, or vitamin K agonists; direct oral anticoagulants remain investigational in children with limited published data on their efficacy [10]. Nevertheless, choosing to treat with anticoagulation is recommended for any symptomatic DVT in pediatric patients; the choice of drug, dose, and duration remains greatly dependent on their presentation and risk factors [10].

This case report sheds light on an unusual presentation of a DVT in a 16-year-old basketball player who experienced unexplained leg pain following open reduction and internal fixation of a displaced tibial tuberosity fracture and a closed reduction internal fixation of the radius and ulna. The aim of this report is to emphasize the importance of clinical vigilance in identifying and managing DVT in young patients, where the risk may be less anticipated. Prompt diagnosis and intervention are key in such cases, illustrating the critical role of the healthcare team in ensuring favourable outcomes.

## Case presentation

**Figure 1:**
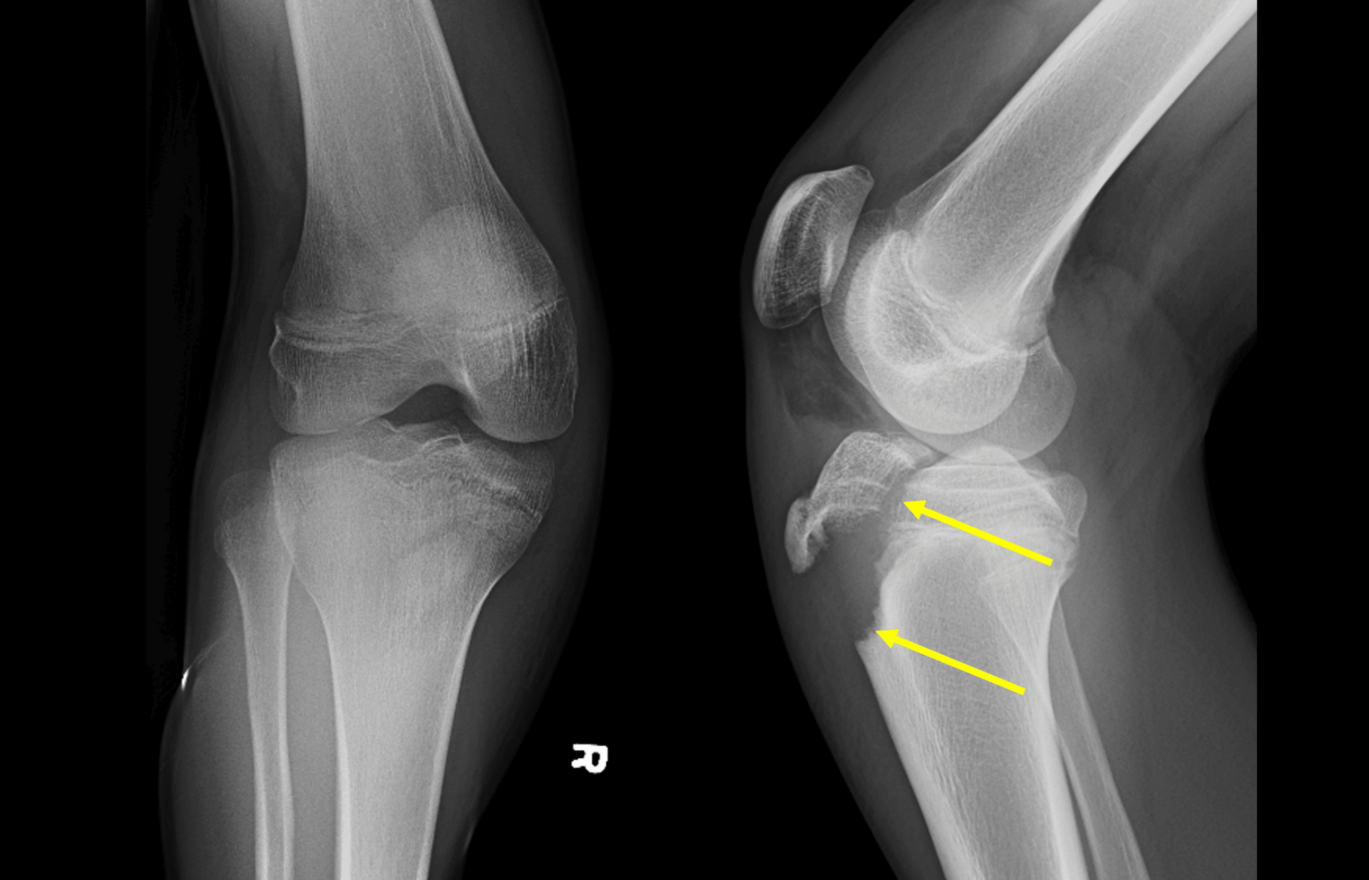
Anterior-posterior (AP) view (left) and lateral view (right) X-rays of the right knee obtained upon presentation to the emergency department. The lateral X-ray highlights an Ogden Classification type 3 tibial tubercle fracture, indicated by two arrows.

The patient is a fit 16-year-old boy, only known for asthma and a penicillin allergy. He has no relevant familial history and no history of substance use. He presented to a tertiary medical center emergency department following a polytrauma basketball injury. While undergoing competitive tryouts for a city team, the patient slipped without contact, awkwardly landing on his right leg and right arm. X-rays confirmed a right tibial tuberosity fracture (Figure 1) and a right ulna and radius transverse midshaft fracture.

**Figure 2:**
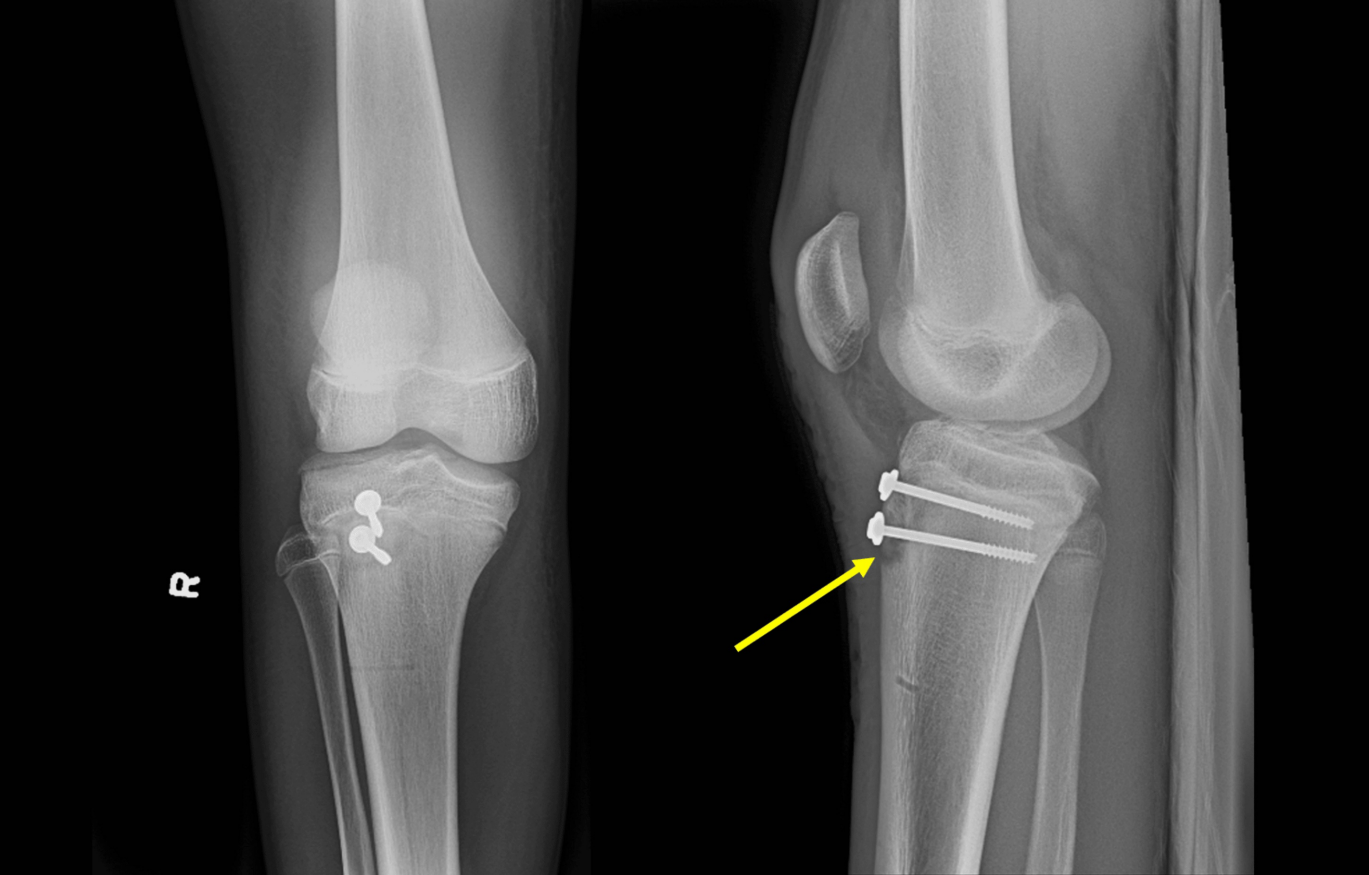
Post-surgical anterior-posterior (AP) view (left) and lateral view (right) X-rays demonstrating the reduced tibial tubercle fracture. The fracture site is indicated by an arrow.

A day later, under general anesthesia, he underwent an uncomplicated open reduction-internal fixation surgery for the tibial tuberosity fracture (Figure 2) along with a closed reduction of the radius and ulna. Both limbs were immobilized with casts.

**Figure 3:**
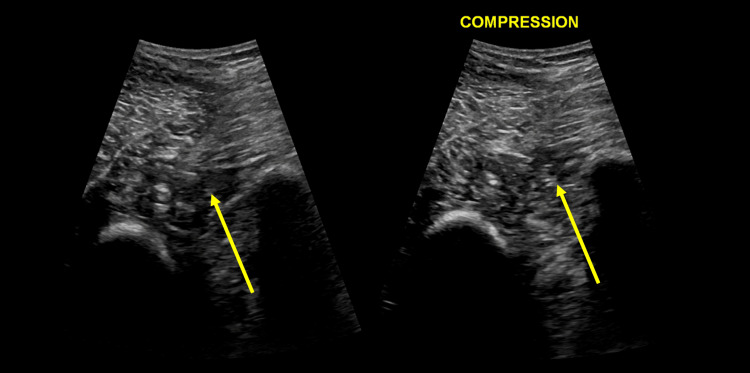
Doppler venous ultrasound of the right lower extremity, focusing on the fibular-posterior tibial vein bundle at symptom onset. Left image: Cross-sectional view showing the fibular and posterior tibial veins, appearing anechoic under ultrasound. Right image: Under forced compression with the ultrasound probe, incomplete compression of the fibular and posterior tibial veins is observed, indicated by the arrow. Proximal veins were compressible with adequate flow.

Four days post-operation, he developed a fever accompanied by increasing severe pain in his right calf, characterized by a burning and pulling sensation. The cast was replaced with a Zimmer splint (Zimmer Biomet, Warsaw, USA). Compartment syndrome was ruled out, and other infectious signs were not present. Doppler venous ultrasound of the lower extremity revealed the presence of an infrapopliteal DVT of the fibular and posterior tibial veins (Figure 3). His Caprini VTE score was 17 [11], and following his symptoms his Well’s score for DVT was 3 [12]. His blood tests are shown in Table 1.

**Table 1:** Relevant patient serum values at the time of symptom onset. FEU: Fibrinogen equivalent units

Test	Value	Units	Reference range
White blood cell count	15	10^9^/L	4.50-11.00
CRP	210	mg/L	0.00-5.00
D-Dimer	1.16	μg/mL FEU	0.00-0.46
Fibrinogen	6.67	g/L	1.50-4.40

Therapeutic enoxaparin was subsequently given at 1 mg/kg, adding up to 60mg, every 12 hours subcutaneously. Intravenous cefazolin was also initiated to treat possible infective thrombus. His lower limb cast was changed to a Zimmer splint to alleviate pressure and visualize the leg. His pain, fever and lab values gradually subsided to their normal ranges over the coming days. He also began mobilizing in his Zimmer splint around his room. Nine days post-DVT, a subsequent venous Doppler identified no evidence of residual infrapopliteal venous thrombosis. He was switched to a prophylactic dose of enoxaparin at 40mg a few days before his eventual stable discharge.

**Figure 4:**
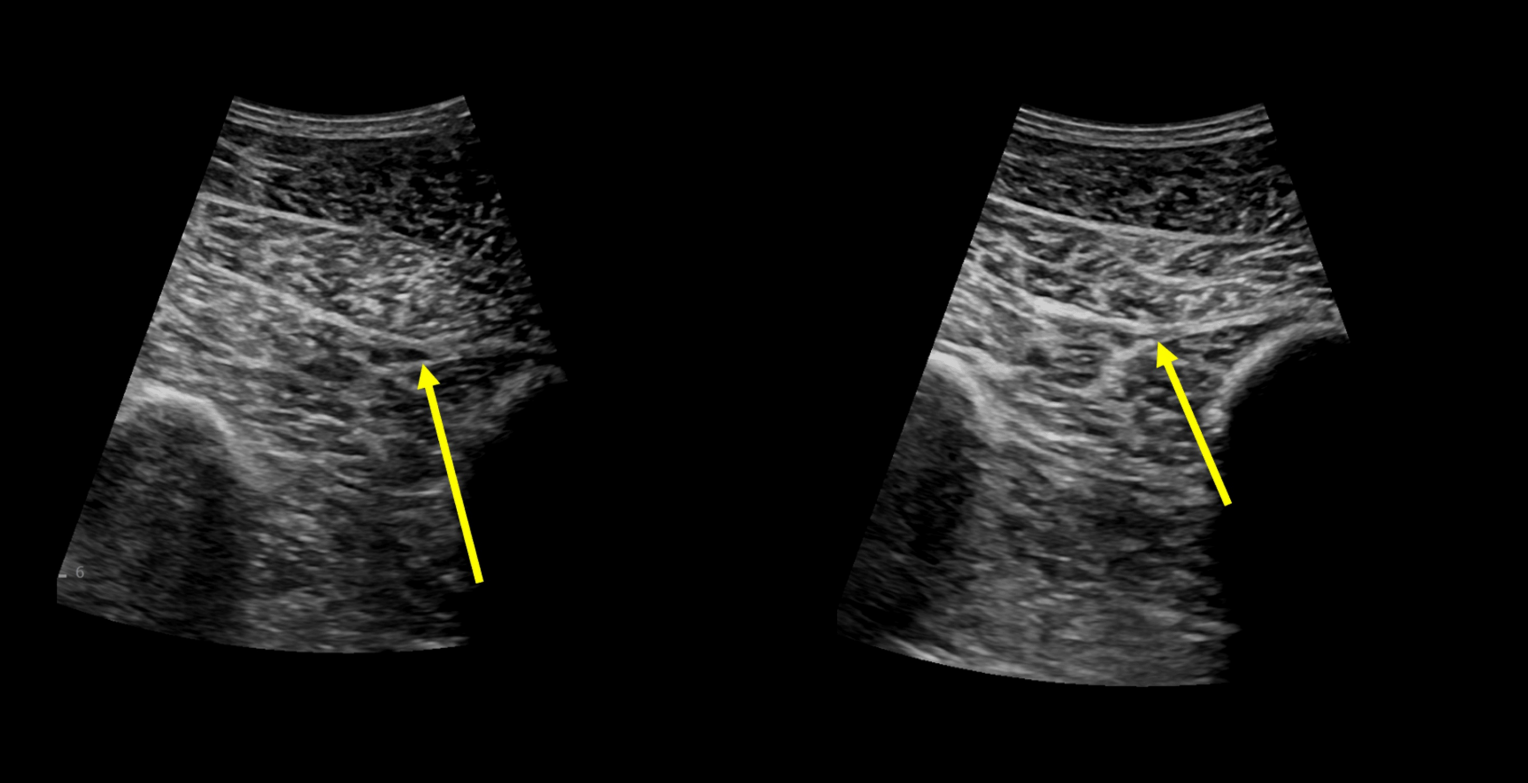
Doppler venous ultrasound of the right lower extremity, 18 days post-treatment. Left image: The veins are less distended compared to baseline, with no compression observed by the probe. Right image: Compression with the probe collapses the veins, with no evidence of residual thrombosis.

A final ultrasound, 18 days post-treatment, showed no recurrence of any DVT (Figure 4). His forearm cast was removed after six weeks and he was no longer wheelchair-bound. His enoxaparin and cefazolin were continuously given until the six-week mark, when he became more active with physiotherapy, and the need for DVT prophylaxis was no longer indicated. He continues to follow hematology for the assessment of a post-thrombotic syndrome.

The patient was out of school during his senior years for more than three months as a result of his injury. He has a long road ahead of him to return to competitive sports.

## Discussion

This case, initially a straightforward and relatively uncomplicated orthopedic injury, evolved into a complex scenario with DVT, causing a difficult time for a young, budding athlete, off school and sports for a lengthy period. His thrombotic risk factors were not many, an open orthopedic surgery with poly-fracture, immobilization post-operatively with casting and an equivocal infection, most of which are present in many of the orthopedic patient populations.

Risk stratifying scores for VTE/DVT, such as the Well’s and Caprini scores, were relatively high in this case; however, a recent study showed the Well’s and Caprini scores had low performance in identifying hospitalized orthopedic children being at increased risk of DVT events [13], significantly limiting their utility in clinical practice. Interestingly, combining a D-Dimer test with the Well’s score showed a mild increase in sensitivity [13]. Having a low threshold for D-Dimer tests post-operatively in polytrauma orthopedic pediatric patients could help increase the identification of a DVT.

Although technically still a pediatric patient, the patient’s nearing adulthood could have increased his risk. One study showed the risk of VTE following trauma was low, with mild increases at 13 years of age, then increasing to the level of adults at 16 years onwards [14].

The use of thromboprophylaxis, on the other hand, is not usually indicated in pediatric populations. Despite some groups suggesting indicators or scores for thromboprophylaxis following pediatric orthopedic surgeries such as in Odent et al., they persisted that prophylaxis should not be used in isolated or polytrauma in the absence of other risk factors in pediatric patients [2].

Ultimately, the timely and effective management of DVT in this case highlights the crucial role of clinical awareness and sound judgement within the healthcare team. The availability of immediate venous Doppler access also facilitated prompt intervention. Had this complication been overlooked or delayed in diagnosis, a severely morbid or fatal risk of pulmonary embolism could have occurred for a patient with his future ahead of him. With an increase in sports injuries referred to orthopedic trauma services, it is imperative for all medical and para-medical staff to maintain vigilant DVT monitoring practices despite reduced risk factors compared to other orthopedic patient populations.

## Conclusions

This case was a routine orthopedic pediatric trauma reconstruction, with few unique risk factors for a possible thrombotic event. Conventional screening tools or risk stratifying scores are significantly limited in their utility due to their low sensitivities, and ultimately, clinical awareness and sound judgement are what will save a patient with a rare and unexpected DVT from a possible morbid or mortal outcome.

It is recommended that future clinical researchers investigate the prevalence of such complications in this growing patient group. This will aid in providing evidence-based guidance on the risk of recurrence, treatments, and safe return to sports plans, enabling informed decision-making.
